# Incorporating cash-based interventions into food assistance programs in humanitarian settings

**DOI:** 10.3389/fpubh.2023.1035554

**Published:** 2023-02-22

**Authors:** Juliette Lash, Anjali Nair, Brittany Markarian, M. Claire Greene

**Affiliations:** Mailman School of Public Health, Columbia University, New York, NY, United States

**Keywords:** cash-based interventions, food assistance, humanitarian response, refugees, nutrition

## Introduction

Food insecurity is on the rise and the most significant increase is seen in conflict and humanitarian settings (1). There are 26 million refugees globally, with as many as 80% facing food insecurity irrespective of location (2). Most refugees reside in low- and middle-income countries where access to food, arable land, and other resources to fulfill basic needs are already limited. As the rate of population growth and the rates of forced displacement have increased, land has become less available. This coincides with changing social dynamics that influence the welcoming of refugees (3). The placement of refugee settlements on land used by host communities for agriculture and the lack of clarity around the boundaries of refugee settlements has caused conflict between these communities (2, 4). These challenges and others have led to policy and guidance advocating for an approach to a humanitarian response that strengthens services and infrastructure for both the refugee and host community and bridges these efforts with larger-scale community development initiatives (3, 4). Cash programs for food assistance have the potential to expand to local markets and build economic security for both refugee and host populations while meeting the nutritional and basic needs of these communities.

## History of food aid and cash programs

The largest international food aid organization, the World Food Programme, was created in 1961 to provide food to emergency-affected communities with its first response being in 1962 after an earthquake struck Buin Zahra, Iran (5). Food distributed in this response consisted of basics such as wheat, sugar, and tea. While providing food assistance was a critical aspect of these responses, the nature of humanitarian emergencies and opportunities to innovate the way support is provided to emergency-affected and displaced populations are evolving. One consistent theme emerges in the current humanitarian sector: that history and the way we “used to do things” is insufficient as the crises the world is experiencing today are increasingly challenging, protracted, and complex.

Traditional food assistance is no longer sufficient in the current humanitarian context. The increasingly protracted nature of humanitarian crises and the large increasing numbers of refugees have forced the humanitarian sector to rethink how it delivers food assistance to refugees and other displaced populations (6). The current humanitarian system, agencies, and donors are overwhelmed by the increase in need and complexity of humanitarian emergencies. This, coupled with the lack of political will from nations to create sustainable programs has led to a system that can no longer function effectively without reconfiguration (7). Food assistance is a central component of humanitarian response that provides short-term assistance for basic needs but puts little attention on addressing the underlying causes of food insecurity. The United Nations High Commissioner for Refugees (UNHCR) recommends moving away from immediate traditional camp-based operational responses such as providing basics (e.g., wheat, rice, sugar, tea etc.) to more development and long-term responses that address underlying issues (6). Cash-based interventions fall under this category and are becoming more common in the humanitarian sector (6).

A cash-based intervention is an intervention in which cash or vouchers for goods or services are provided to a population in need (8). Some examples of these interventions are solely providing cash, allowing refugees to choose between cash or food parcels, supplementing food distribution with cash, or the incorporation of cash in livelihood-building initiatives (8). Cash-based interventions can be delivered physically or digitally through ATMs, bank notes, mobile/e-money, debit cards, or value vouchers redeemable at local markets (6, 9). These forms of cash may be distributed *via* direct cash transfers, cash for work, conditional cash transfers, or voucher programs for a bundle of goods (10). Cash-based interventions and other livelihoods and integration-focused strategies have been proposed as solutions to combine resources across the humanitarian and development sector. Development organizations first utilized cash assistance as a social protection investment to reduce vulnerability as well as increase access to resources (10). Seventy percent of food assistance provided by the World Food Program (WFP) globally went to protracted and complex emergencies with 95% of assistance delivered in direct cash in addition to food assistance (11, 12). The Fill the Nutrient Gap (FNG) analysis, conducted by the World Food Program, has placed cash programs as a focus in their research on nutrition in refugee settings as an identified intervention that can decrease food insecurity and improve nutritional status of refugees (9). These solutions have the potential to more efficiently address the multiplicity of needs in protracted emergency settings as traditional food aid programs often have only short-term benefits.

## Impacts of cash-based interventions

Cash-based interventions have the potential to influence a range of public health outcomes, but evidence on their impacts is mixed in low-income and middle-income settings where aid is given (13–15). Evaluations of the World Food Program's delivery of cash assistance in conjunction with food parcels in refugee camps have revealed that offering refugees the choice to receive cash in lieu of food parcels yield dietary improvements, higher diet diversity, increased financial stability and food security, and greater feelings of self-sufficiency (11, 12, 16, 17). Multiple evaluations of cash-based programs have also demonstrated improvements in height, HAZ scores, and newborn birth weight in addition to positive pregnancy outcomes across age groups and geographical contexts (18, 19). Despite these proximal indicators of nutritional improvements, one study evaluating a cash-based intervention of internally displaced persons in Somalia did not find evidence that cash programs reduced the risk of acute malnutrition (20).

Other non-nutritional impacts identified in evaluations of cash-based interventions in refugee settings include improved psychological wellbeing, which has been associated with food security in observational studies (16, 21). Empirical evidence on the impact of cash-based interventions on other health outcomes is sparse; however, simulation studies have not supported that replacing traditional food assistance with cash led to meaningful changes in a range of chronic diseases and other health outcomes. Rather, this study argued that supplementing traditional food parcels with more fruit and vegetables could have a greater impact on reducing the incidence of chronic diseases and mortality (22). More empirical studies are needed to understand the mechanisms and impacts of cash on health outcomes. Additionally, findings regarding the impact of cash-based interventions on healthcare-seeking behaviors are mixed, suggesting that cash programs need to be integrated into a broader public health strategy to promote healthcare access and utilization (16, 23, 24).

Cash-based interventions may also have an impact on general humanitarian operations. A 2011 study on food security and humanitarian assistance among displaced Iraqi populations in Jordan and Syria identified the importance of cash programs, particularly in protracted crises in urban settings where refugees have been integrated among host populations (10). In both Jordan and Syria, cash assistance was provided to Iraqi refugees and was targeted based on vulnerability criteria (e.g., Syria's eligibility criteria for beneficiary selection: female-headed households, unaccompanied minors, presence of a disabled household member, adults age 60+ not accompanied by an adult male of working age, and families individually assessed to require financial assistance). This study found that cash and voucher programs compared to large-scale food distributions were associated with lower logistical costs and more flexibility for refugees.

In addition to these logistical and functional advantages, cash programs bring dignity, empowerment, and self-sufficiency to refugees. In a qualitative study conducted in Jordan and Lebanon, Syrian refugees reported feeling that cash-based interventions enabled them to meet additional needs and feel as though they were “like a normal citizen” and could integrate within the host population, while also improving dietary diversity and other nutritional outcomes (25). Refugees have expressed they have more variety of foods and are able to meet other needs beyond food (25). A scoping review of food insecurity interventions in refugee settings found that cash-based interventions decreased maladaptive coping strategies (e.g., eating less, removing kids from school) among refugees and the most important effect of cash reported by refugees was eating better while also promoting dignity, sense of safety, increased wellbeing, and reducing intrahousehold tension (2). Gender dynamics and women's safety and wellbeing in refugee camps have always been a large concern for humanitarian response and have emerged in the dialogue surrounding the potential unintended harm of cash-based interventions. An evaluation of cash-based interventions by the World Food Program in Uganda did not find evidence of increases in gender-based or domestic violence and found that these programs equally benefit male and female-headed households (17). Furthermore, implementations of cash-based interventions have not shown an increase in general violence in refugee camps nor an increase in violence related to the increase in women's decision making as cash is generally given to females in the household (17).

## Spillover of cash-based interventions on hosts and local economy

Evaluating the impact of food assistance, including cash-based programs, requires adopting a whole-of-society approach to understand the total impacts, both direct and indirect, on refugee and host populations. Refugees and host populations are not homogeneous; therefore, understanding the indirect impacts of cash programs and other forms of food aid requires contextualization.

One argument for cash programs is that they may support the local economy. When given food rations, refugees may choose to sell them in local markets. Eighty-nine percent of refugees sell some of their food rations for below market price (26). While simulation models generally support that cash programs for refugees have a greater economic impact on host communities than food (26), other studies have found that whether these programs improve economic outcomes for host communities depends on the stage of the emergency (e.g., early stages of the influx vs. protracted displacement context) as well as the relative socioeconomic status of individuals within the host community. Refugees receiving cash have expressed they have access to more food compared to traditional humanitarian-provided food parcels (25).

Researchers have argued that services should be provided comprehensively to reach refugee and host populations. Overall economic development creates demand for labor, higher wages, better supply of goods and services, the ability to pay for services for cash programs to benefit refugees and host communities, and to avoid amplifying disparities between and within these populations (3). Even more specifically, “better-off” host communities can benefit while poorer host communities may be negatively affected (3). Benefits and costs to host communities include food, land, labor, wages, services, common property resources, and economic development (3). In addition to these economic and labor market benefits, including the host community in food and nutrition interventions may improve the relationship between the refugee and host population (2).

## Discussion

Cash programs, in conjunction with traditional food assistance, may strengthen humanitarian response efforts, bring dignity and empowerment to refugees, and spill over into host communities (11). Some critics express concern that cash-based programs may not consistently demonstrate expected impacts on nutrition and other public health outcomes. There are several potential factors that may contribute to this inconsistency. First, there is heterogeneity in how cash-based programs are designed and implemented. There is also significant heterogeneity in how nutritional status and outcomes are measured and evaluated (27). Outcomes are often clinically focused (vitamins or anemia) which may not capture the full picture of the nutrition status of a population and vary greatly based on the humanitarian context in which food assistance and cash-based interventions are delivered. Furthermore, similar to food parcel distribution, there are general guidelines and standards for delivering cash to displaced communities that may not be appropriate for certain contexts or environments.

More research and investment into when, how, and for whom cash programs can be most effectively delivered may clarify some of the mixed evidence that currently exists and improve humanitarian operations to meet the long-term needs of refugees, host communities, and other emergency-affected populations.

## Author contributions

JL led the conceptualization, research, and writing of the article. JL, AN, BM, and MG contributed to manuscript writing and revisions. All authors approved the final version of the manuscript.
